# Supplementary material for: The Relationship between Dog Ownership, Psychopathological Symptoms and Health-Benefitting Factors in Occupations at Risk for Traumatization
Source: Int J Environ Res Public Health. 2020 Apr 8;17(7):2562. doi: 10.3390/ijerph17072562 (PMC7178020; doi:10.3390/ijerph17072562)
Supplement: Supplementary file 1 [file ijerph-17-02562-s001.pdf]

International Journal of
Environmental Research and Public Health

MDPI

Supplementary Material A

The Relationship between Pet Ownership, Psychopathological Symptoms and Health-benefitting Factors in Occupations at Risk for Traumatization

Johanna Lass-Hennemann°[1], Sarah K. Schäfer°[2], M. Roxanne Sopp[3] and Tanja Michael[4,*]

Saarland University, Department of Psychology; 66123 Saarbrücken, Germany; sarah.schaefer@uni-saarland.de (S.K.S.); roxanne.sopp@uni-saarland.de (M.R.S.); t.michael@mx.uni-saarland.de (T.M.)

° Shared first authorship

* Correspondence: lass-hennemann@mx.uni-saarland.de; Tel.: +49-681-302-71011 (JLH)

1. Results for the Comparison Between Dog/Cat Owners and Others

1.1. Demographic Group Differences Between Dog/Cat Owners and Others

Sample characteristics of dog/cat owners and others are presented in Table A-1. Dog/cat owners and others were not different regarding the proportion of women, χ^2 (1) = 3.12, p = 0.077, age, t (575) = 1.24, p = 0.214, and job experience, t (544) = 0.55, p = 0.586. However, dog/cat owners were more likely to live together with a partner or family as compared to non-dog owners, $\chi^2(1) = 10.76$, $p = 0.001$. Moreover, dog/cat owners and others did neither differ with respect to their frequency in working shifts, $\chi^2(1) = 1.61$, $p = 0.204$, nor to the proportion of those working night shifts or on standby duty.

Table 1. Sample characteristics for dog/cat owners and others.

	Dog/cat owners	Others	χ^2/t(df)	p
Sex (% women)	44.70	37.40	$\chi^2(1) = 3.12$	0.077
Age (in years)	38.89 (11.19)	37.68 (11.80)	t (575) = 1.24	0.214
Form of living (living alone, %)	16.3	27.8	$\chi^2(1) = 10.76$	0.001
Job experience (in years)	16.99 (11.10)	16.44 (11.87)	t (544) = 0.55	0.586
Shift work (%)	63.0	57.8	$\chi^2(1) = 1.61$	0.204
Night shifts (% of those working shifts)	82.60	85.50	$\chi^2(1) = 0.55$	0.495
Standby duty (% of those working shifts)	36.60	29.00	$\chi^2(1) = 2.04$	0.154

Note. df = degree of freedom.

Numbers in brackets indicate standard deviations or degrees of freedom.

1.2. Group Differences: Psychopathological Symptoms

General mental health problems. A t-test for independent samples with group (dog/cat owner vs. others) as independent variable and GSI scores as dependent variable revealed no significant group difference, t (569) = 1.07, p = 0.285, d = 0.09. Moreover, there were no moderating effects of gender, F (1,567) = 0.25, p = 0.616, $\eta^2_p = 0.00$, and age, F (1,564) = 0.18, p = 0.672, $\eta^2_p = 0.00$. Furthermore, it did not impact on the findings if individuals live alone or together with a partner or family, F (1,567) = 0.09, p = 0.766, $\eta^2_p = 0.00$.

Posttraumatic stress symptoms. A t-test for independent samples with dog/cat owner versus others as independent variable and IES-R total scores as dependent variable did not show a significant between-group difference, t (496) = 0.09, p = 0.931, d = 0.01. As for general mental health problems, there were no moderating effects of age, F (1,491) = 0.01, p = 0.913, $\eta^2_p = 0.00$, and form of living, F (1,494) = 1.32, p = 0.250, $\eta^2_p = 0.00$. However, respondents' gender showed an impact on PTSD symptom levels: Women reported higher levels of PTSD symptoms, F (1,494) = 6.99, p = 0.008, η^2_p =

0.02. Moreover, there was a significant interaction of dog/cat-ownership and gender, F (1,494) = 4.03, p = 0.045, η^2_p = 0.01. Post-hoc comparisons showed that among non-dog/cat owners women and men did not differ in PTSD symptoms, t (287) = 0.48, p = 0.643, d = 0.06, while women reported more severe PTSD symptoms in the group of dog/cat owners, t (207) = 3.12, p = 0.002, d = 0.43.

Burnout symptoms. A MANOVA with dog/cat owner versus others as between-subject factor and burnout symptoms (emotional exhaustion, depersonalization, and personal accomplishment) as dependent variable did not result in significant group differences, F (3,567) = 0.06, p = .983, η^2_p = 0.00. Moreover, there was no significant moderator effect of gender, F (1,565) = 0.09, p = .964, η^2_p = 0.00, age, F (1,562) = 0.25, p = 0.859, η^2_p = 0.00, and form of living, F (1,565) = 1.04, p = 0.374, η^2_p = 0.01.

1.3. Group Differences: Health-Benefitting Factors

Sense of coherence. A t-test for independent samples with dog/cat owner versus others as independent variable and SOC scores as dependent variable did not show significant group differences, t (578) = 0.34, p = 0.731, d = 0.03. Furthermore, there were no moderator effects of gender, F (1,576) = 0.60, p = 0.440, η^2_p = .00, age, F (1,573) = 1.04, p = 0.309, η^2_p = 0.00, and form of living, F (1,576) = 1.84, p = 0.175, η^2_p = 0.00.

Trait-resilience. In line with the findings on SOC, a t-test for independent samples with trait-resilience as dependent variable did not reveal significant differences between dog/cat owners and others, t (576) = 0.85, p = 0.398, d = 0.07. As for SOC, there were no significant moderating effects, gender: F (1,574) = 0.77, p = 0.380, η^2_p = 0.00, age: F (1,571) = 0.23, p = 0.636, η^2_p = 0.00, form of living: F (1,574) = 0.60, p = 0.440, η^2_p = 0.00.

Locus of control. A MANOVA with dog/cat owner versus others as between-subject factor and external as well as internal LOC as dependent variables also did not result in a significant between group difference, F (2,577) = 0.21, p = 0.809, η^2_p = .0001. Moreover, we did not find moderator effects, gender: F (1,575) = 0.48, p = 0.622, η^2_p = 0.00, age: F (1,572) = 2.62, p = 0.074, η^2_p = 0.01, form of living: F (1,575) = 2.46, p = 0.086, η^2_p = 0.01.

© 2020 by the authors. Licensee MDPI, Basel, Switzerland. This article is an open access article distributed under the terms and conditions of the Creative Commons Attribution (CC BY) license (http://creativecommons.org/licenses/by/4.0/).
